# Corrosion Failure Analysis of Nickel-Plated Tubing in CO_2_-Ca^2+^-SRB Environment of Offshore Oil Fields

**DOI:** 10.3390/ma18174006

**Published:** 2025-08-27

**Authors:** Hui Zhang, Shuo Yang, Kongyang Wang, Chuang Song, Jinyang Hu, Xiaoqi Yue

**Affiliations:** 1 State Key Laboratory of Offshore Oil and Gas Exploitation, Beijing 102209, China; zhanghui7@cnooc.com.cn (H.Z.); wangky11@cnooc.com.cn (K.W.); songchuang3@cnooc.com.cn (C.S.); hujy8@cnooc.com.cn (J.H.); 2 Tianjin Branch, CNOOC (China) Co., Ltd., Tianjin 300451, China; 3 Institute for Advance Material and Technology, University of Science and Technology Beijing, 30 Xueyuan Road, Beijing 100083, China; ys872512917@163.com

**Keywords:** nickel plating, corrosion failure, SRB

## Abstract

Corrosion failure of oil well tubing in the ocean can lead to significant economic losses. Surface treatment is often used to enhance the corrosion resistance of tubing, while corrosion acceleration will occur in a certain environment. This work combined onset failure analysis and corrosion simulation measurements to understand the failure procedure and corrosion mechanism of nickel plating materials in calcium chloride water-type weak corrosion environment. The microscopic analysis results of the failed part show CO_2_ corrosion products co-deposit with SRB bacterial sulfide products and Ca compounds. The damage of nickel plating is accompanied by S-containing products, which was confirmed by simulated immersion experiments at 50 °C, 0.28 MPa CO_2_ partial pressure, and a speed of 3 m/s. The aggressive solution penetrates through the micro-damage pores, followed by the degradation of the Ni plating layer into NiS, leading to the localized loss of protection and triggering under-deposit corrosion. Concurrently, the SRB’s anaerobic environment generates CO_2_ corrosion byproducts and SRB-derived FeS.

## 1. Introduction

Petroleum resources remain extensively utilized as vital strategic commodities. With continuous advancements in offshore oilfield exploration, encompassing both horizontal expansion and vertical depth progression, the corrosion resistance performance of oil well tubular components operating in complex marine environments has emerged as a critical focus in contemporary petroleum engineering research. Even in environments conventionally considered mildly corrosive—such as those containing low concentrations of Cl^−^ [1], dissolved carbon dioxide CO_2_ [2], and H_2_S [3]—materials may still undergo corrosion failure due to synergistic interactions between marine factors and corrosive species. Hu et al. [4] investigated the galvanic corrosion behavior of offshore platform steel in marine thermocline environments, where this corrosion form acts jointly with seawater corrosion on the steel. In industrial production, low-chromium steel and stainless steel with specific content are typically used [5], or surface treatments are applied to enhance the corrosion resistance of steels. Kumar et al. [6] deposited hybrid polyurethane/polypyrrole composite coatings on 316 stainless steel to enhance its corrosion resistance in saline media. Mouez et al. [7] demonstrated that depositing superhydrophobic carbon nanotube coatings on maraging steel reduced the surface corrosion current density by three-fold and enhanced corrosion resistance by five times compared to bare steel. For low-yield oil and gas wells where the service life of tubing does not exceed 5 years, surface-treated carbon steel offers superior cost-effectiveness.

Owing to its elevated position in the galvanic series, nickel is acknowledged in materials science as an alloy component imparting both exceptional corrosion resistance [8,9,10] and excellent mechanical properties [11], while nickel-based alloys demonstrate outstanding corrosion resistance under oilfield service conditions [12,13,14]. Furthermore, the corrosion resistance of nickel-based alloys can be further enhanced through various processing techniques. In their research, Xing et al. [15] improved the mechanical properties and corrosion resistance of nickel alloys fabricated by arc additive manufacturing through the addition of TiC particles. Javidparvar et al. [16] enhanced the tribological properties and corrosion resistance of a cast nickel–aluminum bronze alloy by depositing copper-doped, diamond-like carbon (Cu-DLC) coatings. Cheng et al. [17] studied the synergistic effect of Ni and Cr in improving the corrosion resistance of rail steel.

Although the addition of nickel can effectively resist sweet corrosion, its high cost limits the application of nickel-containing tubing in low-production oilfields. In contrast, employing nickel-based surface treatments presents a more economical alternative. Nickel platings are highly regarded due to their excellent corrosion resistance, uniform deposition thickness, and superior wear resistance [18,19]. Common fabrication methods for nickel platings include electroless plating, electrochemical deposition, and physical vapor deposition. Among these, electrochemical deposition offers the advantages of uniform and controllable plating thickness, low cost, and suitability for large-scale production. However, the resulting platings often exhibit relatively low hardness and limited adhesion strength [20]. Physical vapor deposition (PVD) techniques are capable of producing platings that are hard, uniform, and dense [21]; however, the relatively high manufacturing cost limits their widespread industrial adoption. In contrast, electroless plating offers a more cost-effective alternative, characterized by broad substrate compatibility and the ability to produce uniform and compact platings. Owing to these advantages, it has been extensively utilized in industrial-scale applications [22].

In marine environments, nickel platings are frequently utilized to protect carbon steel tubing, substantially improving their resistance to pitting and localized corrosion. However, the inherent complexity of such chemical platings may also introduce potential failure risks. Field observations have indicated that, under specific conditions involving low flow rates and mildly acidic media, these platings can, counterintuitively, accelerate the corrosion process. The associated failures are typically manifested as pitting corrosion [23], localized attack, or even perforation. Nevertheless, the fundamental mechanisms underpinning these phenomena under mildly corrosive conditions remain largely unclear and have garnered limited research attention to date.

In this study, we conducted a systematic corrosion failure analysis on a failed section of nickel-coated carbon steel tubing retrieved from a marine oilfield wellbore. By integrating surface characterization techniques with environment-simulated corrosion immersion testing, we identified the critical environmental factors contributing to plating degradation and accelerated corrosion. Furthermore, we elucidated the dominant corrosion mechanisms. The findings of this study provide theoretical insights and technical guidance for optimizing protective plating design and material selection in offshore oilfield applications.

## 2. Failure Analysis

### 2.1. Service Environment

The analysis first focused on a non-failed section of nickel-coated tubing retrieved from the same service zone as the failed component. The tubing was supplied by Tianjin Branch, CNOOC (China) Co., Ltd and fabricated from N80-grade carbon steel, with its full chemical composition detailed in Table 1. The sample test section has a length of 500 mm, an inner diameter of 75.99 mm, and a wall thickness of 6.45 mm. To enhance corrosion resistance, a Ni-P plating was deposited on the inner wall of the tubing via an electroless plating process. During its operational period, this tubing’s surface was subjected to a mildly corrosive environment, influenced by the synergistic effects of several factors. Key contributors included dissolved CO_2_, a high water cut, and the associated elevated liquid-phase flow rate. Based on the extraction and interpretation of on-site production monitoring data, several crucial environmental parameters were identified as potential drivers for the corrosion and degradation of the nickel plating.

**Figure 1:**
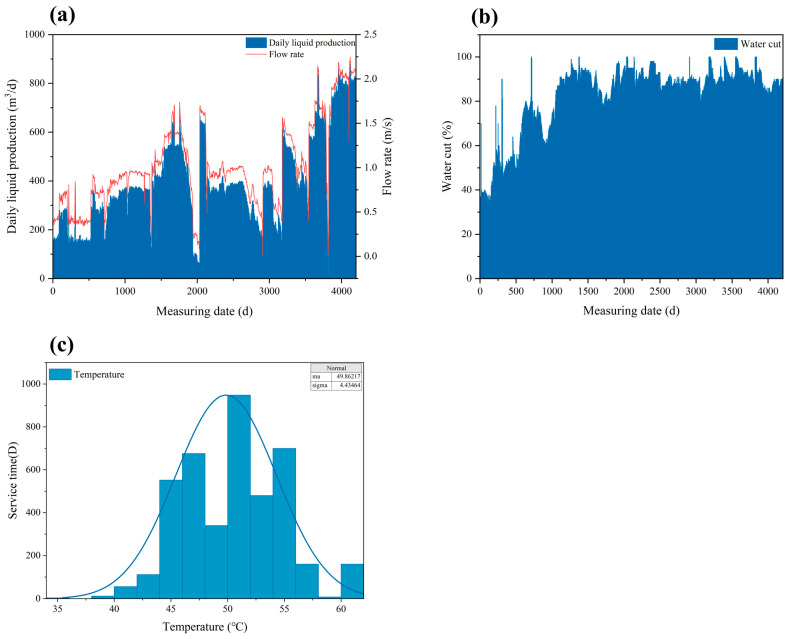
Production operating conditions: (**a**) daily fluid production rate; (**b**) water cut; (**c**) statistical distribution of temperature.

Figure 1a illustrates the variation in daily fluid production over the service period. Early in the operation, the production rate was 150.75 m^3^/d, which gradually increased to 879.36 m^3^/d. This rise in production rate corresponds to an increase in flow velocity within the wellbore from 0.385 m/s to 2.244 m/s, which enhances the mass transfer of corrosive species [24]. This phenomenon may promote flow-accelerated corrosion (FAC), particularly in areas where plating defects are present [25].

Figure 1b depicts the variation in produced water cut over the production period, increasing from 36.8% at the early stage to 100%. This indicates a transition into a high water-cut phase. At this stage, the production fluid within the tubing consists entirely of the aqueous phase, which provides a continuous electrolyte pathway that facilitates electrochemical corrosion processes.

Wellhead parameters were monitored every four days during industrial production, and the recorded data were used to generate Figure 1c, which illustrates the statistical distribution of wellbore temperature for the tubing throughout its service life, from production start-up to corrosion failure. The temperature remained relatively stable, fluctuating between 45 °C and 55 °C, with most of the production time maintained around 50 °C. This specific temperature range is known to be particularly susceptible to CO_2_ corrosion. Within this window, the formation of carbonate species in the aqueous phase is promoted [26], thereby accelerating the localized corrosion of susceptible materials.

The partial pressure of CO_2_ across different service zones within the same aqueous production area was also investigated, revealing a range from 0.083 MPa to 0.277 MPa. This level of CO_2_ partial pressure corresponds to a moderately corrosive environment. In the presence of chloride ions [27], such CO_2_concentrations are known to compromise passive films, thereby facilitating the initiation of pitting and other forms of localized corrosion.

The combined effect of the aforementioned environmental factors creates a complex corrosive scenario characterized by CO_2_ corrosion, high water cut, and fluid shear forces. These factors not only contribute to plating degradation but, when interacting with other conditions, can promote corrosion.

### 2.2. Analysis of Water Samples and Microbial Identification

To further investigate the corrosive environment responsible for plating failure, crude oil samples were collected under normal field production conditions. After allowing the samples to stand at ambient temperature, natural separation of oil and water phases was observed. The upper aqueous phase was extracted for physicochemical and microbiological analysis. These analyses were conducted in the following three main categories: cation analysis, anion analysis, and microbial cultivation.

#### 2.2.1. ICP-OES Analysis of Cations

Inductively coupled plasma optical emission spectroscopy (ICP-OES) was employed to quantitatively determine the major cations present in the water sample. Table 2 summarizes the test results, highlighting a high concentration of divalent calcium ions, which can influence water chemistry by promoting scaling tendencies and enhancing corrosivity [28].

#### 2.2.2. Ion Chromatographic Analysis of Anions

Ion chromatography (IC) was employed to quantitatively determine the major anionic species in the water sample, with the results summarized in Table 3. The moderate concentration of Cl^−^ indicates a corrosive environment capable of disrupting passive films on metal or alloy surfaces, thereby initiating pitting corrosion [27]. The presence of Br^−^ and NO_3_^−^ ions may further enhance localized electrochemical activity, exacerbating the overall corrosion process. These anions are known to contribute to the development of pitting and crevice corrosion on the surface of metallic components [29,30].

#### 2.2.3. Microbiological Cultivation Analysis

To assess the potential influence of microbial activity on corrosion, microbial cultivation was performed using the extinction dilution method in accordance with the Chinese oilfield industry standard SY/T 0532-2012 [31]. Water samples were tested for the presence of typical corrosion-related microorganisms commonly found in oilfield environments, including sulfate-reducing bacteria (SRB), iron bacteria (FB), and heterotrophic bacteria (TGB). Sampling was conducted at three wellheads, designated as A, B, and C, with each group including parallel controls.

**Figure 2:**
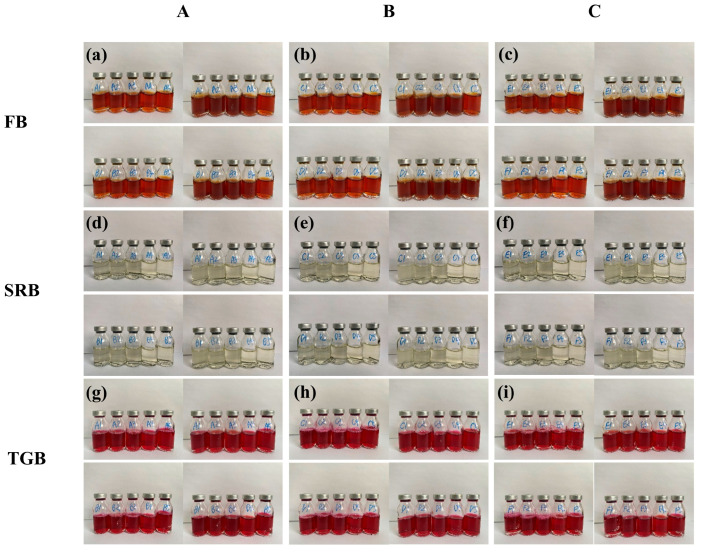
Primary-stage microbial culture results. (**a**–**c**): FB bacterial culture results from three wells (top) and the control group (bottom); (**d**–**f**): SRB bacterial culture results from three wells (top) and the control group (bottom) (**g**–**i**): TGB bacterial culture results from three wells (top) and the control group (bottom).

FB cultures were successfully established, as evidenced by distinct discoloration observed in the media (Figure 2a–c). Specifically, in groups A, B, and C, bottles 1–4 exhibited visible color changes, while bottle 5 remained unchanged. In groups D, E, and F, all five bottles (1–5) showed discoloration. Following dilution-level counting and standard-based calculations, the bacterial concentrations were determined to be 2500 cells/mL, 7000 cells/mL, and 11,000 cells/mL, respectively. In contrast, SRB (Figure 2d–f) and TGB (Figure 2g–i) were not detected during the initial incubation stage (<7 days).

**Figure 3:**
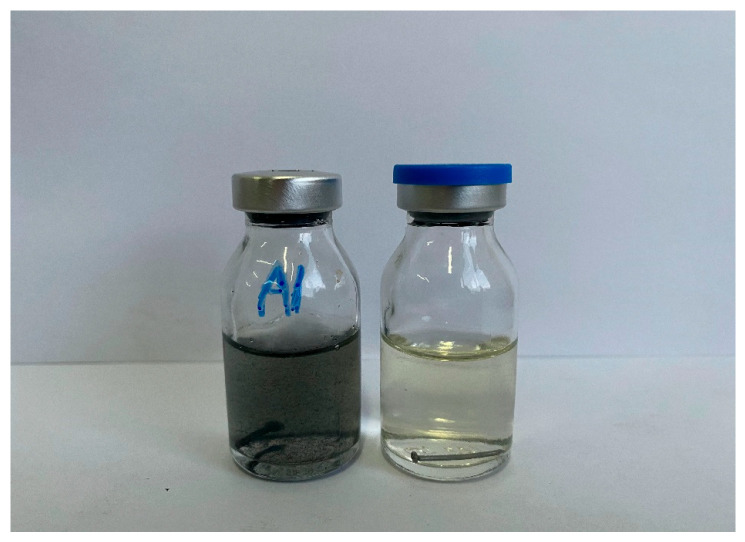
SRB identified in the extended incubation stage.

However, during the extended incubation phase (>7 days), one sample vial tested positive for the presence of SRB (Figure 3). The delayed detection of SRB is attributed to their strict anaerobic nature. It is postulated that residual oxygen in the culture medium initially inhibited SRB proliferation. Over time, this oxygen was likely consumed by aerobic or facultative microorganisms, thereby creating an anaerobic environment conducive to SRB growth and activity [32]. Tian et al. [33] reported that in petroleum reservoir water samples, the abundance of SRB increased significantly only after the oxygen concentration dropped below a critical threshold, even when inoculated under otherwise nutrient-rich conditions. This supports the hypothesis that, during the early stages of incubation, aerobic or facultatively anaerobic microorganisms likely dominate the microbial community, while SRB remain dormant or undetectable until oxygen levels have sufficiently declined. Similarly, the study by Zhu et al. [34] demonstrated that in high-pressure CO_2_-rich transport environments, SRB-induced corrosion was delayed until dissolved oxygen was depleted, with microbial activity gradually shifting from aerobic to anaerobic phases over time. Consistently, the work of Johnston et al. [35] confirmed that oxygen ingress can temporarily suppress SRB metabolic activity, resulting in a delayed onset of sulfide production and associated corrosion. As a well-known causative agent of microbiologically influenced corrosion (MIC) [36], the presence of SRB is of significant relevance to the subsequent analysis of corrosion failure mechanisms.

### 2.3. Microstructural Characterization of the Failed Component

To gain deeper insights into the corrosion mechanisms underlying the failure of the nickel-coated tubing during service, a detailed microstructural analysis was conducted. This investigation primarily encompassed the characterization of corrosion surface morphologies and the examination of the plating cross-sectional structure, employing scanning electron microscopy (SEM) coupled with energy-dispersive X-ray spectroscopy (EDS).

#### 2.3.1. Surface Morphology and Elemental Analysis

**Figure 4:**
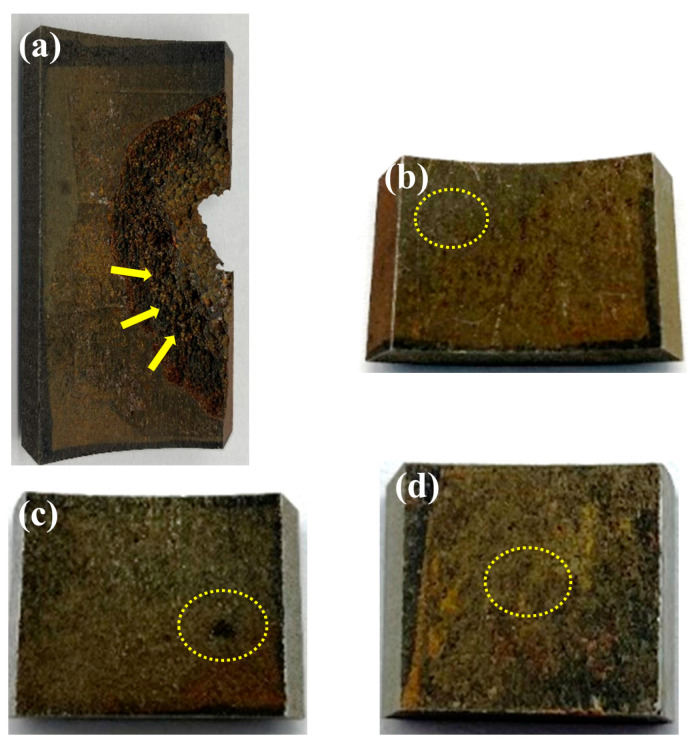
Surface observations of the failed component (marked regions): (**a**) perforation area, (**b**) pitting corrosion area, (**c**) pitting corrosion area, (**d**) localized corrosion area.

Representative areas of the failed component were selected for SEM analysis, including the perforation zone (Figure 4a), pitting corrosion zone (Figure 4b,c), and uniform corrosion zone (Figure 4d).

**Figure 5:**
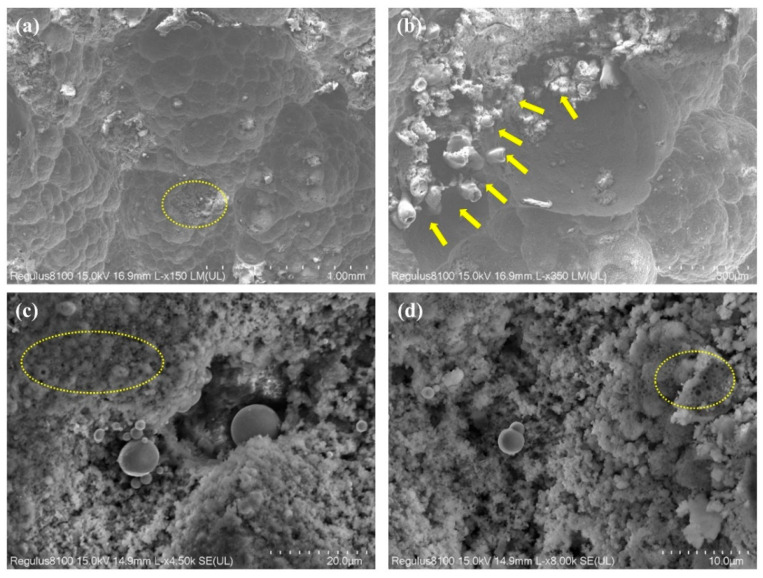
Scanning electron microscopy (SEM) images of the failed component: (**a**,**b**) perforation area; (**c**,**d**) pitting corrosion area.

As shown in Figure 5a, the SEM analysis reveals that the corrosion surface predominantly exhibits typical morphological features of CO_2_ corrosion [37], including extensive dissolution and localized attack. In the perforation and localized corrosion areas (Figure 5b–d), abundant deposits of extracellular polymeric substances (EPS) produced by SRB were observed, indicating microbial involvement in the corrosion process [38].

**Figure 6:**
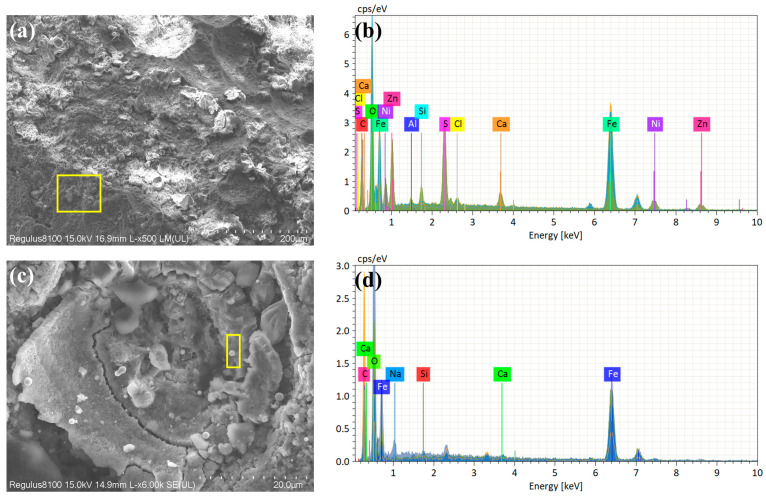
Scanning electron microscopy (SEM) and energy-dispersive X-ray spectroscopy (EDS) (marked region) analysis of the failed component: (**a**,**b**) perforation area; (**c**,**d**) uniform corrosion area.

As shown in Figure 6b, EDS analysis revealed a significant enrichment of sulfur in these regions, accompanied by detectable calcium deposits. In contrast, the scan result from an area with no evident corrosion (Figure 6d) showed a markedly lower concentrations of both S and Ca. These findings are consistent with the service environment conditions described in Section 2.1 (CO_2_-sensitive zones) and the detection of SRB reported in Section 2.2, indicating that SRB play a critical role in the corrosion process.

#### 2.3.2. Plating and Substrate Cross-Sectional Structure Analysis

To further elucidate the plating integrity and failure mechanisms, cross-sectional samples were prepared from both intact plating areas of non-failed tubing and typical corroded regions (including perforation, pitting, and uniform corrosion zones). All samples were mounted using conductive hot-mounting resin with the cross-section oriented upwards. Subsequently, the specimens were sequentially ground and polished using 800-grit and 1000-grit abrasive papers to ensure a smooth and complete cross-sectional surface for microscopic examination.

**Figure 7:**
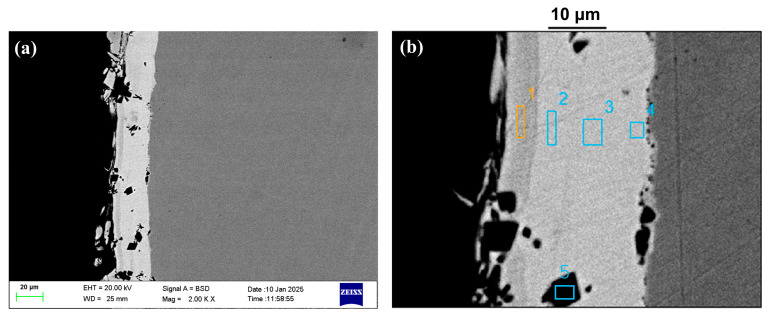
SEM images of the non-failed component: (**a**,**b**) plating cross-sectional morphology; EDS analysis (marked regions 1–5).

In the intact plating region, a characteristic three-layer structure was observed, as shown in Figure 7a. EDS scanning across different layers (Figure 7b) and the corresponding results summarized in Table 4 indicate that the outermost layer is a high-phosphorus sacrificial layer, the middle layer is a dense, wear-resistant nickel layer containing uniformly distributed silicon particles, and the innermost layer serves as a corrosion-resistant nickel barrier. Plating thickness non-uniformity was observed throughout the cross-section, suggesting potential process-related defects during the plating procedure.

**Figure 8:**
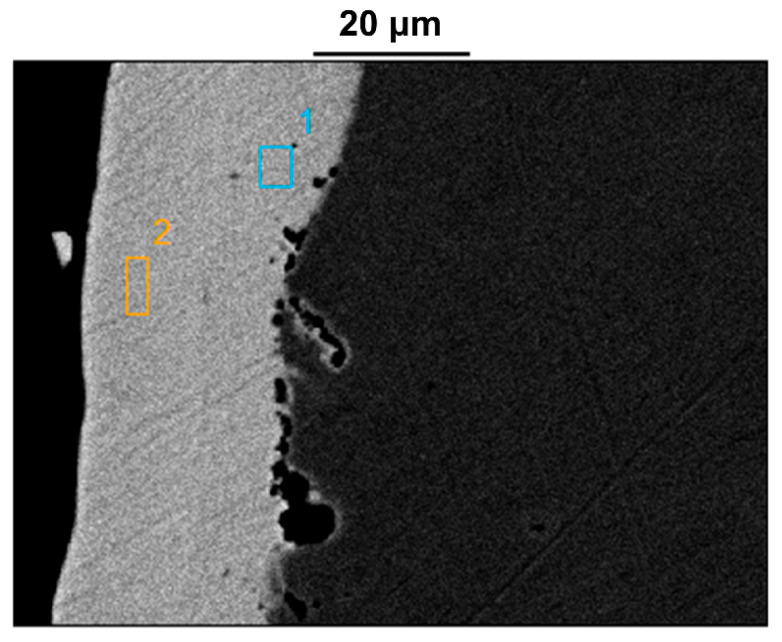
SEM image of the non-failed component—plating cross-section morphology.

As shown in Figure 8, SEM observations of other regions of the intact plating revealed multiple pores distributed along the interface between the plating and the substrate. In some cases, small pores were interconnected, forming larger voids. Analysis of the corresponding EDS results (Table 5) further indicated that, in addition to the non-uniform plating thickness, the deposition process also resulted in compositional inconsistencies in nickel content across the functional layers. This led to the unintended formation of high-nickel corrosion-resistant layers on the outer surface.

**Figure 9:**
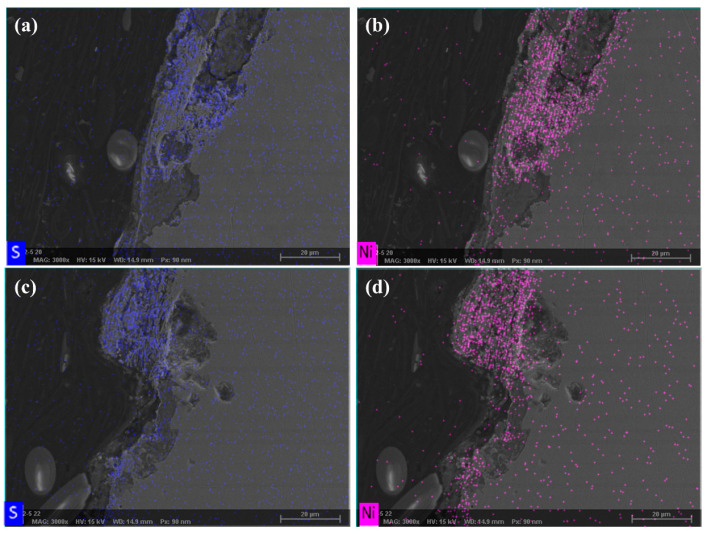
SEM images of the failed component: (**a**–**d**) cross-sectional morphology of the pitting region.

In the failed regions—including the perforation, pitting, and uniform corrosion zones—cross-sectional SEM images reveal the significant structural degradation of the plating. The nickel layer appears thinned or discontinuous, accompanied by the enrichment of S (Figure 9a,c), indicating the SRB in the corrosion process [39]. Notably, corrosion was detected even beneath areas where the nickel plating remains visibly intact (Figure 9b,d), suggesting a loss of protective functionality. These findings imply that the plating’s compactness and barrier integrity have been severely compromised.

The failure analysis revealed that the production fluid contained a high concentration of Ca^2+^ ions, and both SRB and FB were successfully cultured from the water sample. These findings correlate with the presence of EPS observed on the surface of the failed component.

Cross-sectional microstructural analysis of the degraded plating showed the absence of the high-phosphorus sacrificial layer and damage to both the dense wear-resistant mid-layer and the corrosion-resistant inner layer. In the damaged regions, nickel and sulfur signals were highly co-localized, indicating that SRB had converted Ni into sulfide corrosion products, such as NiS. The compromised plating allowed corrosive species, such as CO_2_ and Cl^−^, to penetrate to the substrate surface, initiating under-deposit corrosion. The synergistic effect of CO_2_ corrosion and microbiologically influenced corrosion ultimately accelerated localized failure and perforation of the component.

To further investigate the early-stage failure behavior and mechanisms of CO_2_ corrosion and microbiologically influenced corrosion affecting the nickel plating, corrosion simulation experiments were conducted. These experiments used specimens sectioned from intact, in-service coated tubing retrieved from the field.

## 3. Simulated Corrosion Test

### 3.1. Material and Solution

The material used in this study was N80 carbon steel with a nickel plating, provided by CNOOC (Tianjin) Branch. The nickel plating was applied via an electroless plating method.

Samples were precisely cut into coupons measuring 40 mm in length, 10 mm in width, and 10 mm in thickness, each featuring a 5.5 mm diameter through-hole for mounting. To prevent corrosion of the carbon steel substrate, the remaining five surfaces and the inner wall of the through-hole were coated with high-temperature ceramic adhesive. These coated samples were then dried in an oven at 65 °C for 120 min. Subsequently, they were rinsed with acetone, ethanol, and deionized water, followed by drying with a hot air gun. The prepared samples were stored in a desiccator before being placed into the autoclave. All corrosion experiments were conducted in a C-276 alloy dynamic autoclave.

Based on the analysis of water samples collected near the failure region, two representative solution compositions were obtained, with some differences in calcium and chloride ion concentrations. The composition of Solution A is provided in Section 2.2, while the concentrations of Ca^2+^, Cl^−^, and HCO_3_^−^ in Solution B are listed in Table 6. Prior to use, both solutions were deoxygenated for 24 h. Once prepared, these solutions were mixed with the field-collected water sample at a 9:1 ratio and introduced into the autoclave to simulate microbial factors. The system was maintained under a CO_2_ partial pressure of 0.28 MPa, at a temperature of 50 °C, and with an agitation speed of 3 m/s. To replicate the harsh corrosive environment, nickel-coated specimens were securely affixed using PEEK fixtures within the reactor.

High-pressure CO_2_ was first introduced to the autoclave for preliminary deoxygenation. After this initial purge, a secondary deoxygenation was performed by flushing with medium-pressure CO_2_ twice. Finally, the system was adjusted to the field-representative CO_2_ partial pressure of 0.28 MPa, and the temperature was set to 50 °C. All pressure and temperature parameters were maintained within target tolerances by a precision control system. The specimens were then subjected to an immersion period of 5 days. Upon completion of the exposure, the samples were retrieved and analyzed for surface morphology, cross-sectional features, and corrosion rate.

### 3.2. Results

#### 3.2.1. Degradation of Ni-Plating on Substrate

Localized corrosion rate analysis was conducted by Olympus laser confocal microscope. During the confocal measurement, to minimize height variations caused by the inherent curvature of the tubing, scanning was performed along the longitudinal direction of the tubing. Additionally, multiple sampling areas were selected to reduce measurement errors.

**Figure 10:**
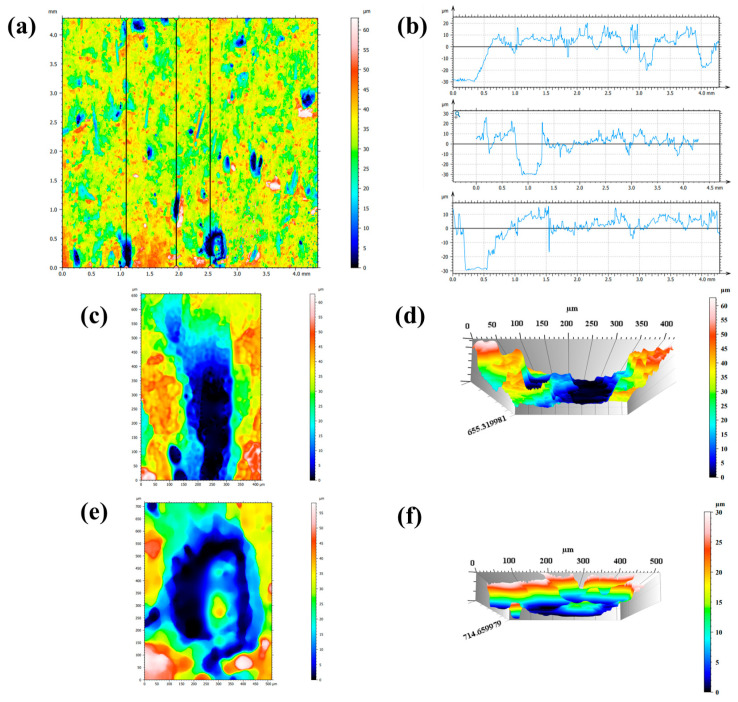
Confocal microscopy results before immersion: (**a**) scanning plane map; (**b**) cross-sectional data analysis; (**c**–**f**) regional extraction and 3D modeling.

Before the experiment, the plating surface of the samples was examined using laser confocal microscopy, and cross-sectional data were extracted for analysis. The scanning plane results are presented in Figure 10a, with a surface porosity of approximately 25.3% having been determined. Cross-sectional analysis was performed based on the selected data (Figure 10b), and in combination with 3D imaging (Figure 10d,f), multiple distinct plateau regions were observed on the sample surface, each approximately 30 μm in thickness—consistent with the nominal plating thickness. These observations indicate the presence of plating defects that compromise the complete protection of the substrate, thereby allowing corrosive species to directly attack the exposed substrate through these defects.

**Figure 11:**
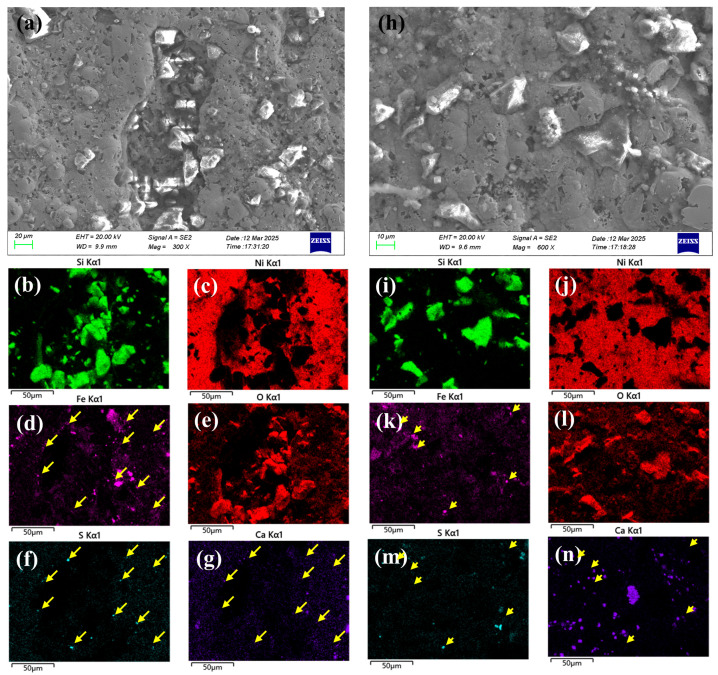
Electron microscopy results: (**a**–**g**) surface morphologies and EDS spectra of damaged plating areas; (**h**–**n**) surface morphologies and EDS spectra of the plating.

After exposure, the plating exhibited significant damage around initial pore, as shown in Figure 11a. The retained wear-resistant silicon dioxide particles from the original plating material at the damaged sites (Figure 11b,c). Pronounced corrosion phenomena were observed at the edges of these damaged areas, as shown in Figure 11d,e, accompanied by sulfide and Ca deposits (Figure 11f,g).

Figure 11h show microscopic pores and defects within the initial compete nickel plating. Note that the Si-rich particles on the top surface are added for wear-resistant purpose, as shown in Figure 11i,j. Within these pores, iron elements were detected (Figure 11k), indicating that the defects extended to the carbon steel substrate, leading to the formation of iron oxide corrosion products (Figure 11l) accompanied by localized sulfur and Ca deposition (Figure 11m,n).

**Figure 12:**
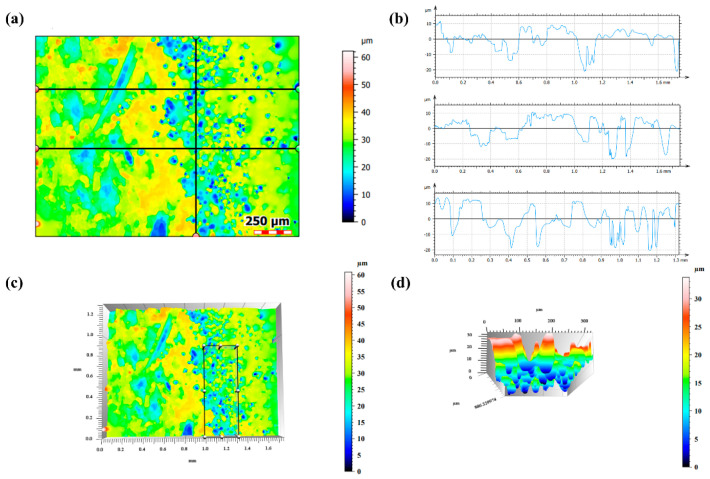
Confocal microscopy results after immersion: (**a**) scanning plane map; (**b**–**d**) cross-sectional data analysis.

The microscopic pores was observed using laser confocal microscopy, and the scanning plane results are shown in Figure 12a. After eliminating the influence of curvature, cross-sectional data were selected for analysis (Figure 12b). The region indicated in Figure 12c was examined using 3D imaging, with localized analysis performed on the pitted areas, revealing multiple discrete corrosion pits (Figure 12d). and the presence of relatively deep pitting. Combined with the scanning data in Figure 10b, the analysis indicated an overall thinning of the plating, with some deeper pits propagating into the substrate, resulting in direct exposure of the base material to the environment. 

**Figure 13:**
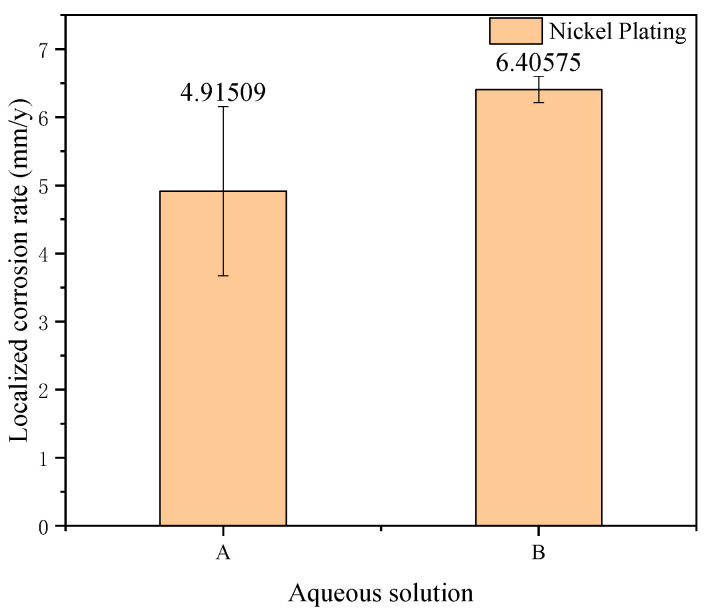
Localized corrosion rate.

The laser confocal scanning data of the samples were analyzed to calculate the localized corrosion rate, as shown in Figure 13. Combined with the thickness data, it can be inferred that perforation of the tubing would occur within two years.

#### 3.2.2. Corrosion of the Substrate Beneath

**Figure 14:**
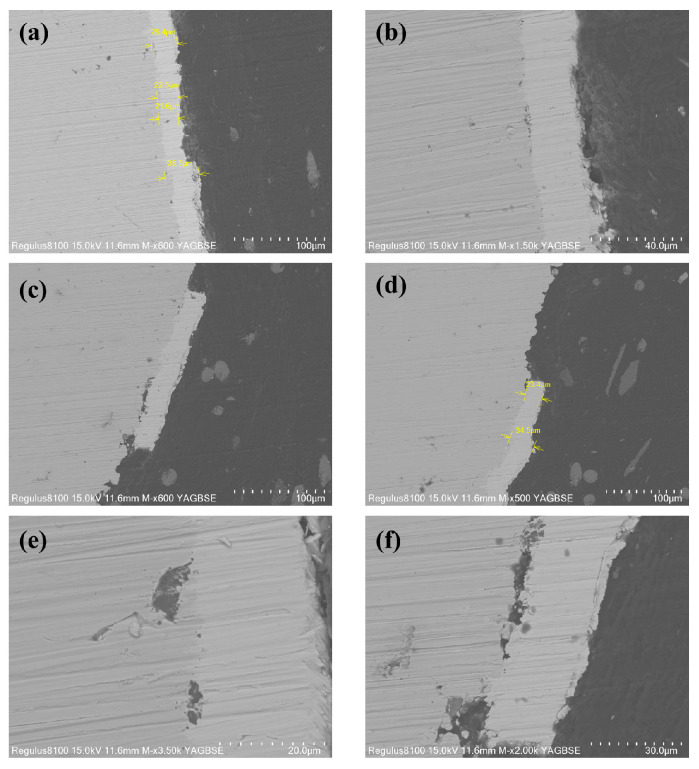
(**a**,**b**) Cross-sectional morphology observations; (**c**,**d**) areas with plating loss; (**e**,**f**) under-plating corrosion.

**Figure 15:**
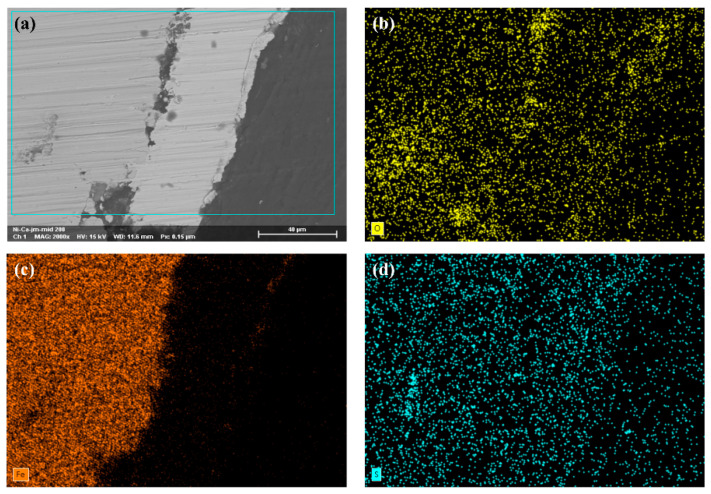
(**a**) EDS scanning area; (**b**–**d**) elemental mappings.

After immersion, the samples were sectioned to prepare cross-sectional specimens. Figure 14 shows that while the plating maintained good integrity in some areas (Figure 14a,b), significant localized damage or complete loss of the coating was observed in others (Figure 14c,d). In these compromised regions, only sparse remnants of the plating adhered to the substrate. Meanwhile, as shown in Figure 14e,f, even in areas where the plating remained present, localized corrosion occurred at the plating–substrate interface, exhibiting a tendency to penetrate deeper into the substrate. During the EDS analysis of the corrosion beneath the coating, a depletion of Fe (Figure 15c) and an enrichment of S and O (Figure 15b,d) were observed. This provides evidence that corrosive media containing high concentrations of Ca^2+^ ions and SRB infiltrated through surface pores in the coating. This process, in turn, created an anoxic environment within the crevice between the coating and the substrate, leading to under-deposit microbiologically influenced corrosion of the substrate.

Biocides are commonly employed to suppress MIC in high-water-cut oilfields, typically during the production process. Compounds such as benzalkonium chloride (BKC) [40], tetrakis hydroxymethyl phosphonium sulfate (THPS) [41], and sodium pyrithione (SPT) [42] have been demonstrated to exhibit bactericidal activity against SRB. However, in practical applications, a substantial increase in biocide dosage is often required to ensure effectiveness. Prolonged and excessive use of biocides not only promotes the development of bacterial resistance but also poses significant risks of environmental contamination in water and soil systems [43].

#### 3.2.3. Electrochemical Testing

**Figure 16:**
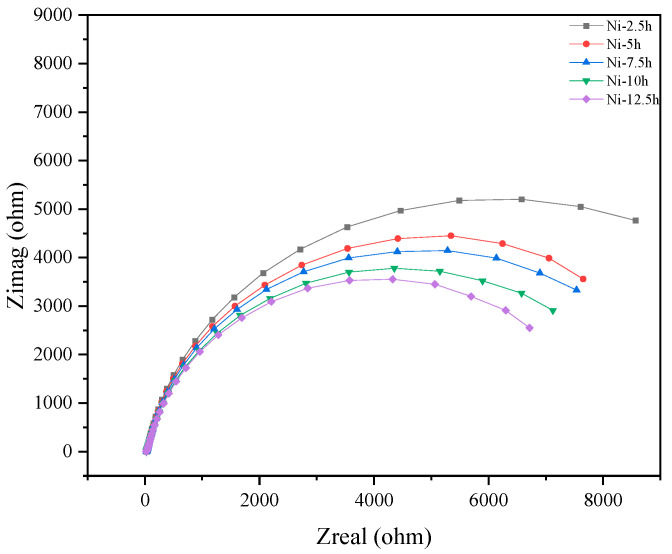
EIS test results.

Electrochemical samples of the nickel plating, measuring 10 mm × 10 mm, were cold-mounted using epoxy resin and immersed in simulated field-condition water. EIS tests were conducted at fixed intervals of 2.5 h. After multiple testing cycles, the EIS results are shown as in Figure 16. Initially, the electrode surface exhibited high impedance, which gradually decreased with increasing immersion time. Notably, significant impedance reduction in the low-frequency region was observed at the 10 h and 12.5 h time points. Repeated testing altered the surface structure of the plating, leading to the formation of pores and changes in porosity. These structural changes enlarged the diffusion pathways of corrosive media and reduced the mass transfer resistance.

## 4. Conclusions

This study aims to investigate the failure mechanism of a nickel coating material in a mildly corrosive environment. Through microstructural characterization and elemental analysis of both unfailed and failed samples, in comparison with simulated corrosion tests replicating field conditions, and in conjunction with an analysis of environmental factors, the internal and external causes of failure were determined.

1. The coating exhibited certain process-related defects, including non-uniform thickness and poor adhesion, along with the presence of pores penetrating through its structure. Corrosion of the substrate was observed even in the adherent regions of the coating, accompanied by an enrichment of S.
2. The surface morphology of the failed component was characteristic of classic CO_2_ corrosion, and cross-sectional analysis revealed a high degree of colocalization between Ni and S. Concurrent with the observation of EPS, and considered alongside the sulfur enrichment and bacterial culture results, it was concluded that the synergistic action of SRB and CO_2_ during service led to the coating’s failure and subsequent substrate corrosion. In light of the challenges associated with chemical biocidal methods, future efforts could be directed toward the development of more sustainable and effective biological control technologies.
3. Following the simulated immersion experiment, the cross-sectional morphology of the coating closely resembled that of the failed component, showing partial detachment. EDS analysis detected substrate corrosion, which was accompanied by the co-enrichment of Ca and S. The pores on the coating surface enabled the infiltration of corrosive media, including fluids with a high concentration of Ca^2+^ ions, to the substrate. Concurrently, an anoxic environment formed at the coating–substrate interface, promoting under-deposit microbiologically influenced corrosion.

## Figures and Tables

**Table 1:** Chemical compositions of the material.

Elements	C	Si	Mn	P	S	Cr	Al	Cu
Comp/wt%	0.31	0.26	1.53	0.01	0.004	0.04	0.04	0.077

**Table 2:** ICP cation test results.

Cation Species	Content (mg/kg)
Ca^2+^	975.50
Mg^2+^	160.15
Na^+^	995.70
K^+^	27.83

**Table 3:** IC anion test results.

Anionic Species	Content (mg/kg)
Cl^−^	5990.40
HCO_3_^−^	152.66
Br^−^	137.78
SO_4_^2−^	45.27
NO_3_^−^	42.00

**Table 4:** EDS elemental composition of the plating on the non-failed component.

Atom (%)	O-K	Na-K	Mg-K	Al-K	Si -K	P -K	Cl-K	K-K	Ca-K	Fe-K	Ni-K	C-K
Pt1	3.0	2.4	1.1	0.2	0.6	14.4	0.2	0.1	0.9	3.3	73.8	
Pt2	2.1		0.3		0.2	9.5			0.3	2.4	85.1	
Pt3					0.0	8.6				2.3	89.2	
Pt4					0.2	7.8				5.6	86.4	
Pt5	42.8				20.6	0.17				0.44	0.39	35.6

**Table 5:** EDS elemental composition of the plating on the non-failed component (marked regions 1, 2).

Atom (%)	P-K	Fe-K	Ni-K
Pt1	9.8	2.9	87.3
Pt2	7.1	1.6	91.3

**Table 6:** Ion concentrations in Solution B.

Cation Species	Content (mg/kg)
Ca^2+^	131.94
Cl^−^	2382.5
HCO_3_^−^	730.90

## Data Availability

The original contributions presented in this study are included in the article. Further inquiries can be directed to the corresponding author.
